# Folding correction of ABC‐transporter ABCB1 by pharmacological chaperones: a mechanistic concept

**DOI:** 10.1002/prp2.325

**Published:** 2017-05-26

**Authors:** Matthias Spork, Muhammad Imran Sohail, Diethart Schmid, Gerhard F. Ecker, Michael Freissmuth, Peter Chiba, Thomas Stockner

**Affiliations:** ^1^ Institute of Medical ChemistryCenter of Pathobiochemistry and GeneticsMedical University of ViennaWaehringerstrasse 10ViennaA‐1090Austria; ^2^ Department of ZoologyGovernment College University LahoreKatchery RoadLahore54000Pakistan; ^3^ Institute of PhysiologyCenter of Physiology und PharmacologyMedical University of ViennaSchwarzspanierstrasse 17ViennaA ‐1090Austria; ^4^ Department of Medicinal ChemistryUniversity of ViennaEmerging Field PharmacoinformaticsAlthanstrasse 14ViennaA‐1090Austria (GFE); ^5^ Institute of PharmacologyCenter of Physiology und PharmacologyMedical University of ViennaWaehringerstrasse 13aViennaA‐1090Austria

**Keywords:** ABC transporter, folding correction, P‐glycoprotein, pharmacochaperone

## Abstract

Point mutations of ATP‐binding cassette (ABC) proteins are a common cause of human diseases. Available crystal structures indicate a similarity in the architecture of several members of this protein family. Their molecular architecture makes these proteins vulnerable to mutation, when critical structural elements are affected. The latter preferentially involve the two transmembrane domain (TMD)/nucleotide‐binding domain (NBD) interfaces (transmission interfaces), formation of which requires engagement of coupling helices of intracellular loops with NBDs. Both, formation of the active sites and engagement of the coupling helices, are contingent on correct positioning of ICLs 2 and 4 and thus an important prerequisite for proper folding. Here, we show that active site compounds are capable of rescuing P‐glycoprotein (P‐gp) mutants ∆Y490 and ∆Y1133 in a concentration‐dependent manner. These trafficking deficient mutations are located at the transmission interface in pseudosymmetric position to each other. In addition, the ability of propafenone analogs to correct folding correlates with their ability to inhibit transport of model substrates. This finding indicates that folding correction and transport inhibition by propafenone analogs are brought about by binding to the active sites. Furthermore, this study demonstrates an asymmetry in folding correction with cis‐flupentixol, which reflects the asymmetric binding properties of this modulator to P‐gp. Our results suggest a mechanistic model for corrector action in a model ABC transporter based on insights into the molecular architecture of these transporters.

**Abbreviations**
ABC: ATP‐binding cassette
HEK293: human embryonic kidney 293 cells
ICL: intracellular loop
MDR: multidrug resistance
NBD: nucleotide‐binding domain
P‐gp: P‐glycoprotein
rh123: rhodamine123
TMD: transmembrane domain

## Introduction

ATP‐binding cassette (ABC) proteins are found in all kingdoms of life and thus form one of the largest protein families (Rees et al. 2009). In humans 48 genes code for ABC proteins, the majority of which are transmembrane transporters. Some members of the human ABC protein family are not involved in active transport, but function in mediating ion conductance or ion channel regulation. Transporters recognize a diverse panel of cargo molecules including lipids, drugs, cyclic nucleotides, nucleobases, and bile acids. Dysfunction of at least 22 human ABC transporters has been linked to more than 27 disease etiologies (Chiba et al. 2014). Tangier disease (ABCA1), progressive familial intrahepatic cholestasis type 3 (ABCB4) and type 2 (ABCB11), Dubin‐Johnson syndrome (ABCC2), pseudoxanthoma elasticum (ABCC6), general arterial calcification of infancy (ABCC6), cystic fibrosis (ABCC7), hyperinsulinemic hypoglycemia of infancy (ABCC8), and gout (ABCG2) are important examples of diseases caused by impairment of ABC proteins. Point mutations, which lead to folding deficiency, are a major cause of diseases associated with ABC proteins. Among them cystic fibrosis is the most frequent with an incidence of approximately 1 in 3500 births in Austria (Farrell 2008).

Several members of the human ABC family are involved in drug disposition. The most intensely studied human multidrug ABC transporter is P‐glycoprotein (P‐gp), also referred to as MDR1 or ABCB1. It exports a diverse panel of structurally and functionally unrelated cytostatic drugs, thereby rendering human cancer cells multidrug resistant. We selected this multidrug transporter as a model system for studying folding because of its ability to interact with a large complement of small molecular weight compounds. This increases the likelihood of identifying active compounds. The mutants ABCB1‐∆Y490 (Hoof et al. 1994), a mutation located in the first alpha helix of the alpha helical domain of NBD1 (recently named the E‐helix in inverted subfamily G transporters) (Lee et al. 2016), and ABCB1‐∆Y1133, its analogous mutation in NBD2, were used in these experiments because amino acid residues in this alpha helix contribute to the ball and socket interaction of ICLs 2 and 4 with the NBDs (Jin et al. 2012). Furthermore, Y490 is the analogous position of F508 in the cystic fibrosis transmembrane conductance regulator (CFTR, ABCC7) (Hoof et al. 1994). Deletion of this residue (∆F508), the most prevalent mutation in cystic fibrosis, is found in at least one allele in 90% of all patients (Kerem et al. 1989; Riordan et al. 1989). The ∆Y490 and ∆Y1133 mutants are representative for frequent other mutations in their vicinity with respect to their position in the alpha helical domain. These include mutations in the breast cancer‐related protein (ABCG2, a major contributor to gout particularly in the Japanese population) (Matsuo et al. 2009), the cholesterol transporter (ABCA1) (Bodzioch et al. 1999; Rust et al. 1999), and the conjugated bilirubin transporter (ABCC2).

Propafenone analogs were extensively characterized by our group in the past and found to be both, substrates and inhibitors of P‐gp (Chiba et al. 1995; Schmid et al. 1999). Active sites were characterized by photolabeling and subsequent identification of ligand‐modified component peptide fragments by mass spectrometry (Pleban et al. 2005; Parveen et al. 2011). ABCB1 is considered to have arisen from a homodimeric ancestor by gene duplication (Gottesman and Pastan 1993). We found that binding of propafenones occurs at the transmembrane domain interface in either of two modes, which are related to each other by pseudosymmetry. During photolabeling the photoactive arylketone group of propafenones is oriented toward the helix 5/8 and 2/11 interfaces respectively. Residues in the latter contribute to the preferred binding mode 2 of these compounds (Parveen et al. 2011). Active site tyrosine residue 953 was identified to contribute to propafenone binding in mode 2 (Donmez Cakil et al. 2014). Previous data demonstrated that substrates and inhibitors of P‐gp can act as pharmacological chaperones by promoting folding of trafficking deficient mutants (Loo and Clarke 1997, 1998, 2013, 2014; Loo et al. 2005). Therefore, we examined the rescue of P‐gp transmission interface mutants ∆Y490 and ∆Y1133 with propafenone analogs, which bind to active sites at the TMD interface, and also with cis‐flupentixol, an allosteric modulator of P‐gp, interaction of which is restricted to TMD2 only. The prediction was that propafenones ought to rescue both mutants, while the rescuing effect of cis‐flupentixol ought to be asymmetric. Indeed such an asymmetry was found. This finding allowed for the development of a mechanistic concept for pharmacochaperone action.

## Materials and Methods

### Knockdown of endogenous P‐gp in human embryonic kidney 293 cells

Human embryonic kidney 293 (HEK293) cells express endogenous P‐gp, which compromises the quantification and functional characterization of transiently expressed P‐gp mutants. Thus, the endogenous protein was knocked down by transduction with pLKO.1 lentiviral vectors containing P‐gp small hairpin (shRNA) constructs targeted to the 3′ untranslated region of the gene as described previously (Donmez Cakil et al. 2014).

### Construction of mutants

Mutants ∆Y490 and ∆Y1133 of hexa‐his‐tagged human P‐gp were generated in the entry vector pENTR4 (Invitrogen, Carlsbad, CA). The following forward and reverse primers were used (Microsynth, Wolfurt, Austria): ∆Y490‐forw 5′‐ GCT GAA AAC ATT CGC GGC CGT GAA AAT GTC ‐3′; ∆Y490‐rev 5′‐ GAC ATT TTC ACG GCC GCG AAT GTT TTC AGC ‐3′; ∆Y1133‐forw 5′‐ GCT GAG AAC ATT GCC GGA GAC AAC AGC CGG ‐3′; ∆Y1133‐rev 5′‐ CCG GCT GTT GTC TCC GGC AAT GTT CTC AGC ‐3′; For transfer of P‐gp constructs from entry vector pENTR4 into the proprietary gateway compatible destination vector pCEP4d, the unique recombination properties of bacteriophage lambda attL and attR sites were used as described (Hartley et al. 2000).

### Expression of P‐gp in HEK293 cells

Transient transfection of HEK293 cells with wild‐type or mutant P‐gp was performed as described previously (Donmez Cakil et al. 2014) using TurboFect transfection reagent (Fisher Scientific, Vienna, Austria).

### Rhodamine123 efflux assay

Rhodamine123 (rh123) efflux assays with transiently transfected HEK293 cells were performed after a 24 h incubation at 28°C or 37°C. Cells were harvested by trypsinization after washing with phosphate‐buffered saline (PBS), and centrifuged at 500 g followed by resuspension of the pellet with DMEM (pH 7.8) containing rh123 (Sigma Chemical Co., St. Louis) at a final concentration of 0.2 *μ*g/mL (0.53 *μ*mol/L). Loading with rh123 was carried out in a water bath under continuous shaking at 37°C for 30 min. Afterwards cell were chilled on ice and subsequently washed with ice‐cold DMEM (pH 7.4) to remove extracellular rh123. Cell pellet was resuspended in DMEM at 37°C (pH 7.4) to start efflux of rh123. The cellular fluorescence was continuously monitored in a Becton Dickinson FACSCalibur flow cytometer (BD Biosciences, Vienna, Austria) and the first order rate constants (*k*‐values) were calculated as described previously (Donmez Cakil et al. 2014).

### Daunomycin efflux inhibition assay

CCRFvcr1000 cells were loaded with daunomycin (Sigma Chemical Co., St. Louis) in RPMI1640 media (pH 7.8) at a final concentration of 1.69 *μ*g/mL (3.0 mol/L), as described for rh123 efflux assay. After washing with ice‐cold RPMI1640 (pH 7.4), cells were resuspended in RPMI1640 media prewarmed to 37°C (pH 7.4) containing either solvent or compound. Eight concentrations (serial 1:3 dilution) were tested for each potential inhibitor. The excitation wavelength for daunomycin was 488 nm and emission was monitored with a 585/42 nm band‐pass filter (FL2). The efflux was measured with a FACSCalibur and IC_50_ values were calculated as outlined previously (Donmez Cakil et al. 2014).

### Quantification of surface expression by indirect immunofluorescence

Transiently transfected cells were cultured at either 28 or 37°C for 24 h for temperature shift experiments. For pharmacochaperone experiments, cells were incubated with compounds or solvent for 24 h at 37°C. Eight concentrations were tested for each compound ranging from 195 nmol/L to 25 mol/L. Subsequently, cells were harvested by trypsinization, washed with PBS, and incubated for 30 min with the primary mouse monoclonal anti‐P‐gp antibody MRK16 (final concentration: 5 *μ*g/mL) (Kamiya Biomedical Company, Seattle, WA) or IgG2a control antibody (final concentration: 2.5 *μ*g/mL) (BD Biosciences, Vienna, Austria). Cells were again washed with PBS and incubated with FITC‐labeled goat antimouse antibody (final concentration: 12.5 *μ*g/mL) (BD Biosciences, Vienna, Austria) for 30 min in the dark at 2–8°C in a cold room. Thereafter, the cells were washed with PBS and resuspended. A Becton Dickinson FACSCalibur flow cytometer was used to measure MDR1 surface expression (Fig. 3A, B). The excitation wavelength was 488 nm and emission was monitored with a 530/30 nm band‐pass filter (FL1). The EC_50_ values for individual compounds were calculated by fitting data points for P‐gp surface expression to the equation of a saturation hyperbola by nonlinear regression.

### Compounds

Cyclosporin A was purchased from Fluka (Sigma Chemical Co., MO). cis‐Flupentixol was a generous gift from Saibal Dey. Propafenones were synthesized as described (Chiba et al. 1995). The structures of propafenone analogs are shown in Table 1. Their authenticity was verified by ^1^H and ^13^C‐NMR.

**Table 1:** Structures of propafenone analogs

Compound	Chemical structure	Molecular mass	IC_50_ of daunomycin transport inhibition Mean ± SD (mol/L)	EC_50_ for rescue of ∆Y490 Mean ± SD (mol/L)	EC_50_ for rescue of ∆Y1133 Mean ± SD (mol/L)	REFERENCE
(R)‐propafenone	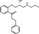	341.44	0.234 ± 0.038	32.63 ± 0.14	37.54 ± 4.77	(Chiba et al. 1998)
(S)‐propafenone	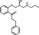	341.44	0.242 ± 0.025	35.95 ± 1.51	40.49 ± 5.52	(Chiba et al. 1998)
GPV005	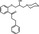	367.48	0.251 ± 0.031	39.39 ± 3.66	38.51 ± 0.60	(Langer et al. 2004)
GPV019	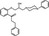	444.57	0.493 ± 0.056	86.36 ± 2.70	89.18 ± 1.23	(Langer et al. 2004)
GPV031	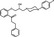	462.56	0.068 ± 0.014	7.03 ± 0.67	5.66 ± 0.85	(Langer et al. 2004)
GPV128	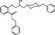	457.60	0.029 ± 0.003	4.01 ± 0.74	4.08 ± 0.17	(Langer et al. 2004)
GPV317	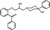	431.52	0.211 ± 0.015	33.65 ± 0.66	38.38 ± 3.23	(Langer et al. 2004)
GPV319	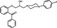	434.50	0.180 ± 0.019	29.43 ± 6.12	25.67 ± 3.72	(Langer et al. 2004)
GPV982	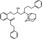	551.76	0.157 ± 0.034	24.92 ± 1.11	26.42 ± 0.27	
GPV984	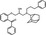	523.70	0.222 ± 0.021	29.22 ± 3.94	30.31 ± 4.43	
GPV987	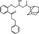	447.61	0.068 ± 0.006	10.87 ± 1.57	11.76 ± 2.60	

### Homology modeling

The ABCB1 homology model was generated as described previously (Stockner et al. 2009). The model of human P‐gp is based on the crystal structure of *M. musculus* P‐gp (PDB ID: 4XWK) (Nicklisch et al. 2016).

## Results

### Selection of P‐gp transmission interface mutants

Crystal structures of several prokaryotic and mammalian ABC exporters share a common architecture, in which each TMD forms contacts with both NBDs via segments, which are referred to as coupling helices. Thereby partially domain‐swapped TMD/NBD interfaces (also referred to as transmission interfaces) are formed, which function in propagating conformational changes from the NBDs to the TMDs and vice versa. These transmission interfaces are frequently targeted by disease‐causing mutations that lead to improper protein folding and subsequent trafficking deficiency (Hashimoto et al. 2002; Imai et al. 2002; Hirouchi et al. 2004; Tornovsky et al. 2004; Hayashi et al. 2005; Cheong et al. 2006; Singaraja et al. 2006; Le Saux et al. 2011; Gautherot et al. 2012; Sorrenson et al. 2013). A systematic review of the literature for naturally occurring single nucleotide polymorphisms (SNPs) and single amino acid deletions was performed. We identified 126 confirmed trafficking deficient mutations in 12 different ABC proteins from four subfamilies. For an alignment of full‐length transporters and homodimeric half transporters, sequences of the latter were duplicated. We were able to assign positions of 85 mutations to the ABCB1 protein sequence. For the ABCA subfamily an alignment of the TMDs was compromised because secondary structure analyses show large differences in the length of transmembrane domain helices. For the ABCG subfamily, the recent publication of the ABCG5/G8 structure indicates a dissimilar TMD fold, with limited alignment to NBDs (Lee et al. 2016).

**Figure 1:**
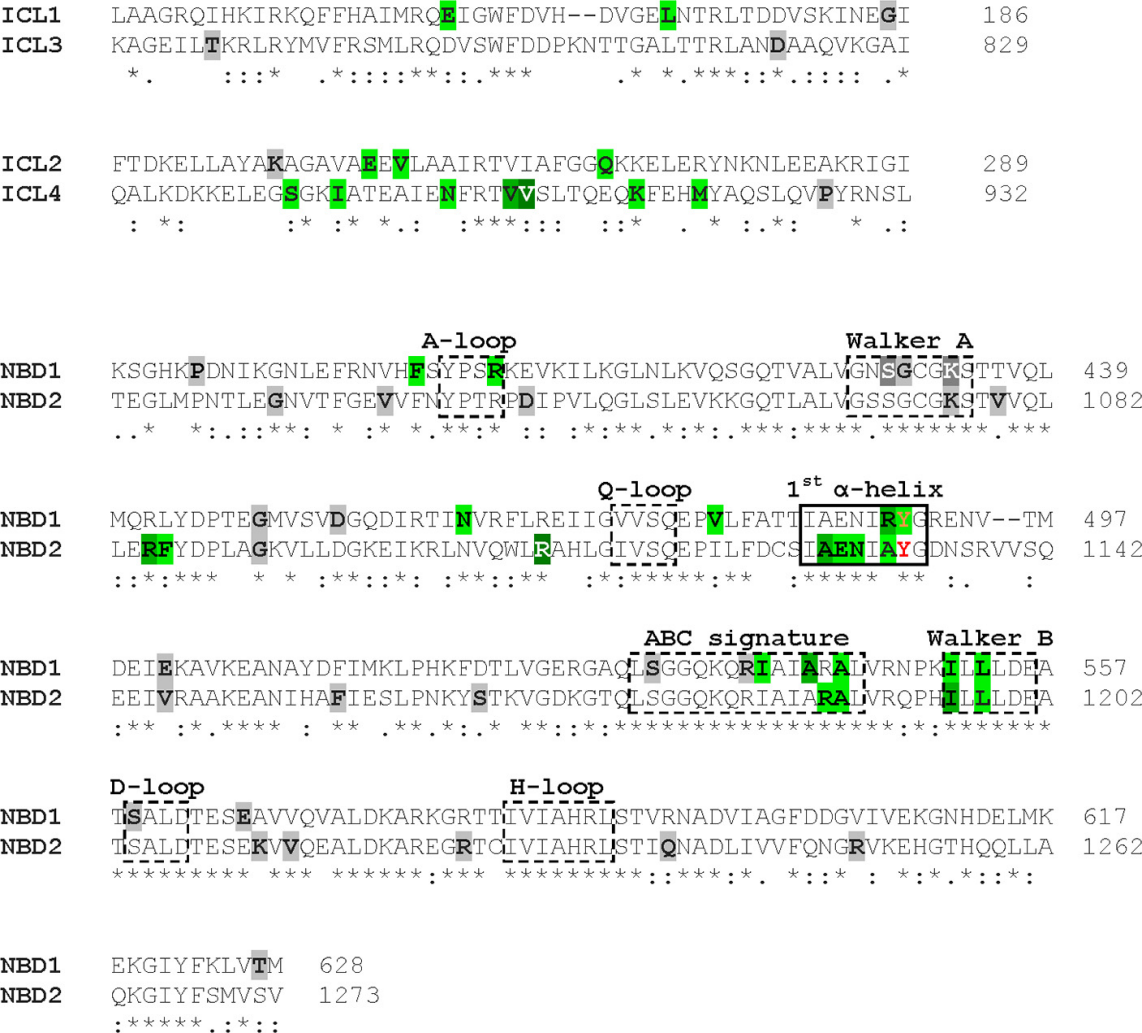
Sequence alignment of N‐terminal and C‐terminal ICLs and NBDs of human P‐gp. Positions of tyrosines 490 and 1133 are indicated in orange and red font, respectively. Furthermore, analogous positions of folding deficient SNPs and single amino acid deletions of human ABC proteins are highlighted in the sequence of human P‐gp. Mutations at the transmission interfaces are highlighted in different shades of green, whereas others are indicated in different shades of grey. Darker colors indicate more than one ABC protein carrying a SNP or an amino acid deletion at the analogous position. Stippled boxes refer to sequence motifs, the black box to the first alpha helix of the alpha‐helical domain (also referred to as the E‐helix in ABCG5/G8). SNPs are also summarized in tabular form in Table S1. SNPs, single nucleotide polymorphisms.

Forty‐one of these 85 mutations were found to be located at the transmission interfaces (15 in TMDs, 26 in NBDs). This distribution is highly nonrandom, given that only a minor percentage of residues is involved in its formation. We further focused on the 26 interface mutations in the NBDs. Mutations in the NBD motifs, as well as those affecting glycine residues – the importance of which for molecular mechanics has been documented – were excluded from further consideration because rescuing nonfunctional mutants would not be a viable therapeutic option. This systematic survey identified a cluster of naturally occurring SNPs and amino acid deletions in the first alpha helix of the alpha helical domains (conforming to the E‐helix in ABCG5/G8) and its C‐terminal loop region (Fig. 1).

**Figure 2:**
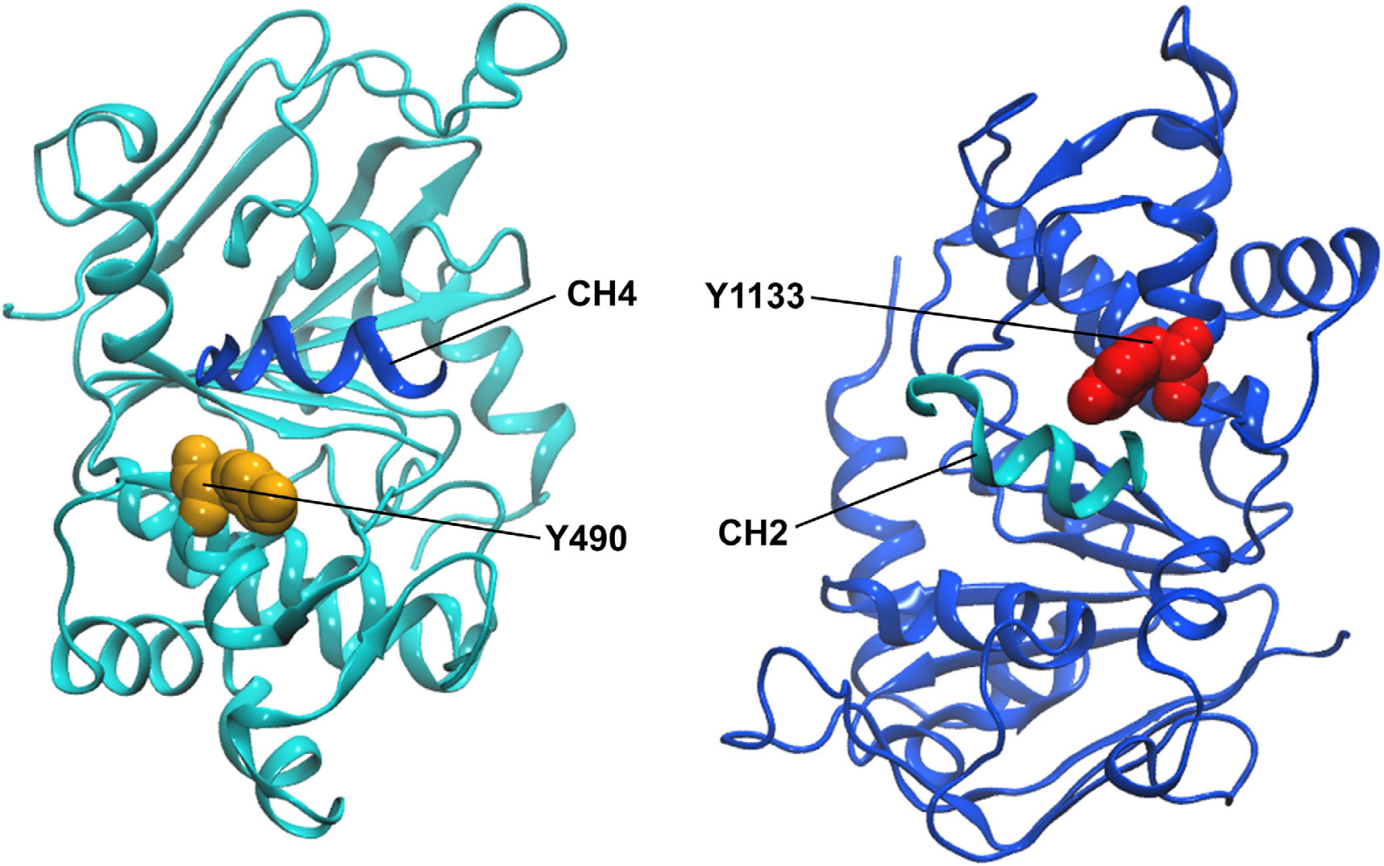
The positions of tyrosine 490 in NBD1 (orange), tyrosine 1133 in NBD2 (red), and coupling helices (CH) of ICLs2 and 4 (CH2, CH4) are depicted in a homology model of human P‐pg (template *M. musculus* P‐gp (PDB ID: 4XWK)). The N‐terminal half of the protein is colored in cyan, the C‐terminal half is depicted in dark blue.

**Figure 3:**
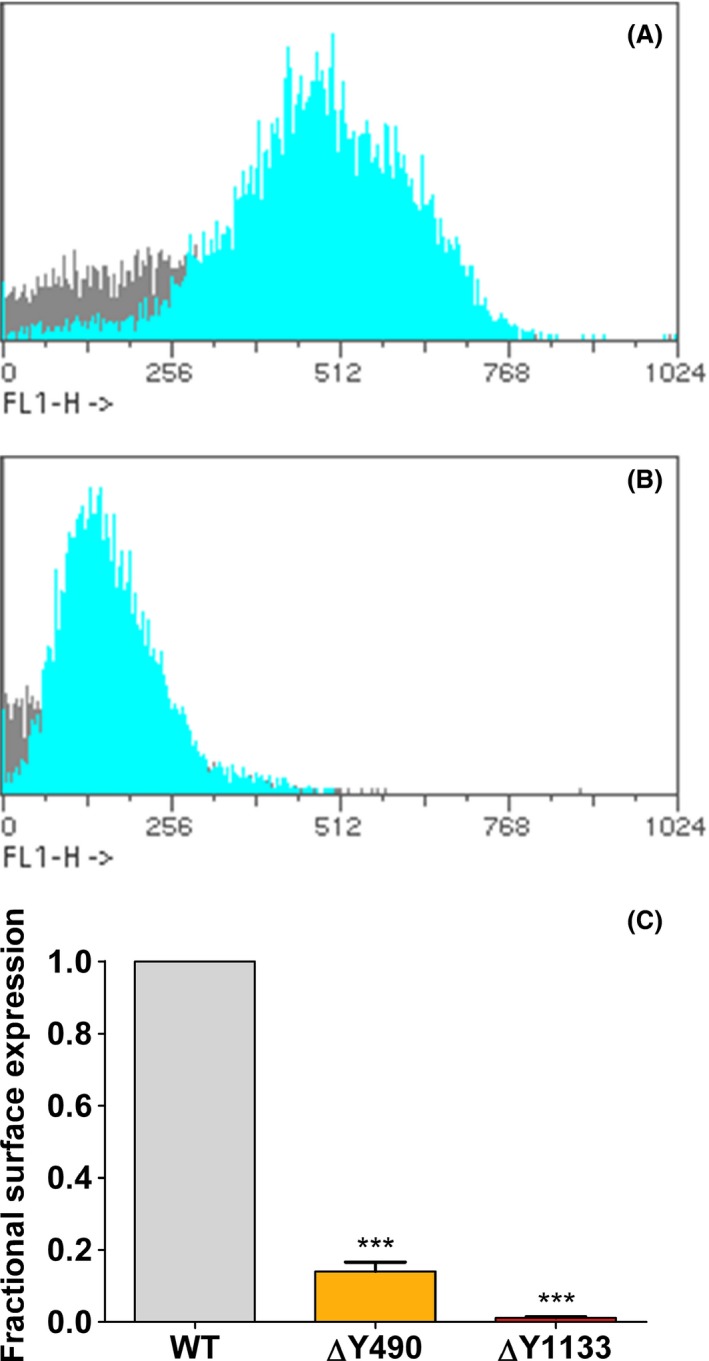
(A) Representative histogram of the surface expression of wild‐type (WT) protein. The MRK16 antibody was used as the primary antibody. FITC‐conjugated goat antimouse antibody was used as the secondary antibody. Fluorescence was measured in a FACSCalibur flow cytometer as described in detail in the methods section. The abscissa shows mean fluorescence intensities (as channel numbers) in FL1. Viable cells are indicated in cyan. The arithmetic mean of the population was determined. From this, the mean fluorescence units per cell were calculated. The latter are a relative measure of P‐gp surface expression. (B) Same as A, but for mutant ΔY1133. (C) Bar graph showing the fractional surface expression of P‐gp interface mutants in comparison with wild‐type (WT) protein. Surface expression was determined by indirect immunostaining with the MRK16 antibody using flow cytometry and normalized to surface expression of WT protein. Means ± S.D. from seven independent experiments were compared using one‐way ANOVA. Post hoc Tukey analyses were performed to find groups with significantly different mean values. Significantly different from wild type: ***P < 0.001.

We generated a mutant of human P‐gp (∆Y490), which is analogous to the paradigmatic mutant ∆F508 in CFTR (Hoof et al. 1994) and also the corresponding mutant in NBD2 (∆Y1133) (Figs. 1, 2 and S1A, B). As expected for a rotationally symmetric protein (Gottesman and Pastan 1993), deletion of tyrosine Y1133 also rendered P‐gp trafficking deficient. Both tyrosine residues are highly conserved for the entire ABCB subfamily with the exception of ABCB8, which carries a phenylalanine residue at the corresponding position. Plasma membrane expression levels of transmission interface mutants were significantly decreased in comparison to wild type (WT). Mutants ∆Y490 and ∆Y1133 showed 14 ± 4% and 1.1 ± 0.4% surface expression, respectively (Fig. 3C). Thus, a significantly lower expression level was found for the ∆Y1133 than for the ∆Y490 mutant. This is in agreement with data from the literature showing that mutations at the NBD2/ICL2 interface affect folding more severely than analogous mutations at the NBD1/ICL4 interface (Loo et al. 2013; Loo and Clarke 2015).

### Effect of temperature shift on interface mutants

**Figure 4:**
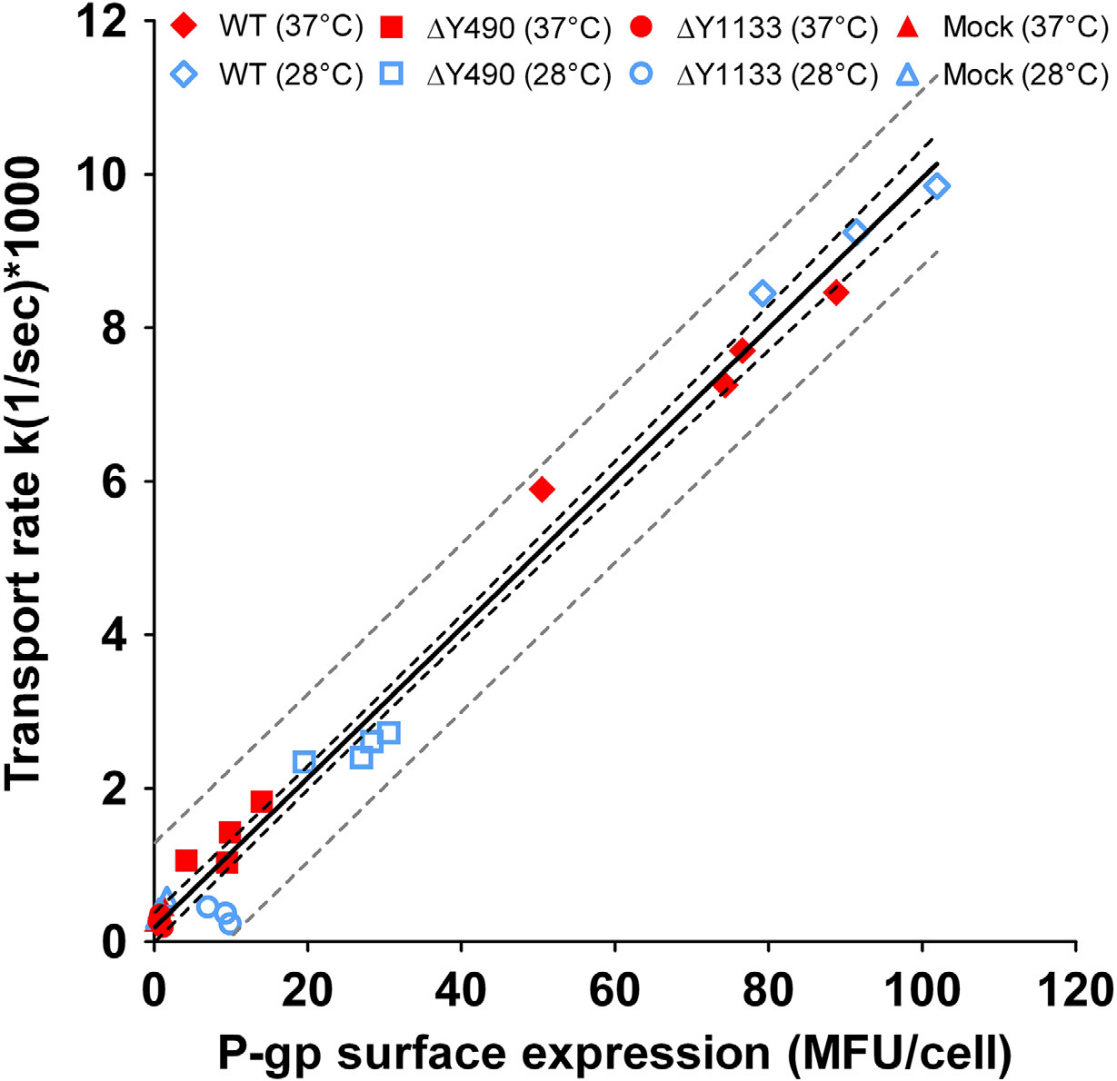
A total of 27 efflux experiments are shown. Data points represent the mean of duplicate determinations. First‐order rate constants of rh123 transport are plotted as a function of P‐gp surface expression (determined by indirect immunostaining with the MRK16 antibody). The square of the correlation coefficient is 0.99 with a *P* < 0.001. A linear regression fit, the 99% confidence interval (black dashed lines), and 99% prediction interval (grey dashed lines) are indicated.

In recent years folding correction by pharmacological chaperones has arisen as a viable concept for the treatment of diseases caused by aberrant folding. Sapropterin dihydrochloride, a first pharmacological chaperone for the treatment of phenylketonuria, has been introduced in the market in the United States in 2007 (Thompson 2008), thus providing a proof of principle that folding correction by pharmacochaperones is a valid therapeutic approach. The concept of folding correction with small molecules is also supported by several in vitro studies on members of the ABC‐transporter family (Polgar et al. 2004; Loo et al. 2005; Pedemonte et al. 2005; Iram and Cole 2012). Importantly, treatment with pharmacological chaperones would only be considered successful if the rescued mutants were still functional. In order to test if transmission interface mutants ∆Y490 and ∆Y1133 were functional when expressed at the plasma membrane, a temperature shift to 28°C was used to promote maturation of the nascent protein (Machamer and Rose 1988). Thermal rescue of folding deficiency has been amply demonstrated and was also specifically reported for human ABC family members (Denning et al. 1992; Loo and Clarke 1994; Plass et al. 2004; Delaunay et al. 2009). Plasma membrane expression levels and first‐order rate constants for rh123 transport were determined 24 h after shifting cells to 28°C. Expression of mutants ∆Y490 and ∆Y1133 increased by a factor of 3 ± 1 and 11 ± 4 (*n* ≥ 3), respectively. Figure 4 shows transport rates as a function of surface expression. For any transporter a linear relationship between these parameters is obtained as long as its function is not impaired. For transport‐deficient mutants slopes were lower than that for wild‐type protein. Data points for wild‐type P‐gp and the ∆Y490 mutant were found to be located on the same line, indicating that the mutant is functional once it reaches the plasma membrane. For the ∆Y1133 mutant low expression levels precluded a definitive statement about its functionality (Fig. 4).

### Rescue of P‐gp mutants with propafenones

**Figure 5:**
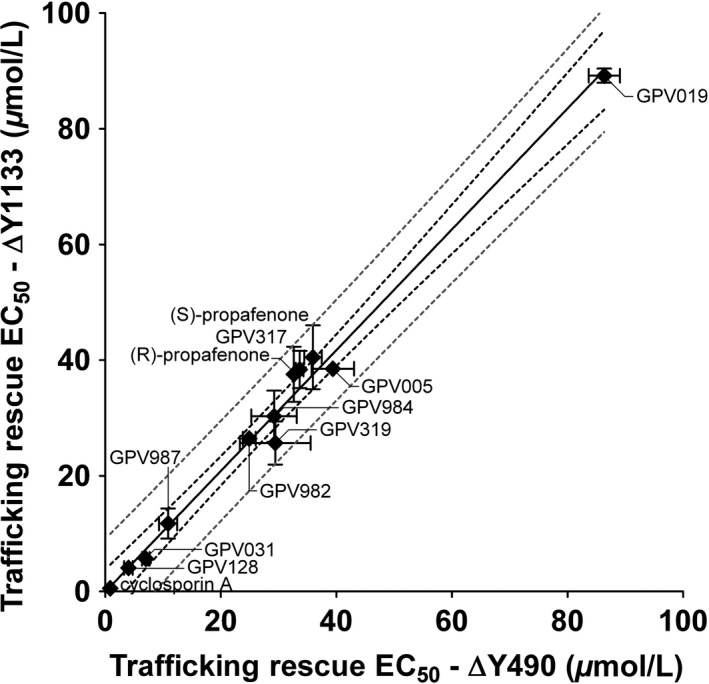
Correlation between EC_50_ values for trafficking rescue of mutants ∆Y1133 and ∆Y490. EC_50_ values were calculated by nonlinear regression analysis of concentration response curves. The square of the correlation coefficient is 0.99 with a *P* < 0.001. A linear regression fit minimizing sum of squares, the 99% confidence interval (black dashed lines), and 99% prediction interval (grey dashed lines) are displayed. Mean ± S.D. of at least two independent experiments.

We extensively characterized propafenone analogs in the past with respect to their properties as both substrates and inhibitors of P‐gp (Chiba et al. 1995; Schmid et al. 1999). Previous studies on P‐gp revealed that substrates and inhibitors can act as pharmacological chaperones (Loo and Clarke 1997, 1998, 2013, 2014; Loo et al. 2005). More than 60 propafenone analogs were screened at three concentrations (0.25, 2.5, and 25 mol/L) and 11 of those were found to be active at least at the highest concentration used (data not shown). For active compounds full concentration–response curves were obtained. Figure 5 shows a comparison of the rescue potential in the two mutants for these 11 propafenone analogs. Chemical structures of those compounds are shown in Table 1. Cyclosporin A was used as a reference pharmacological chaperone (Loo and Clarke 1997). EC_50_ values were calculated by nonlinear regression. Almost identical EC_50_ values for these compounds were obtained for both mutants (*R* ^2^ = 0.99) (Fig. 5). Thus, propafenones have the capacity to rescue folding of P‐gp transmission interface mutants. Rescue is concentration dependent and similar for both the ∆Y490 and ∆Y1133 mutant.

### IC_50_ values for daunomycin transport inhibition and EC_50_ values for trafficking rescue correlate

**Figure 6:**
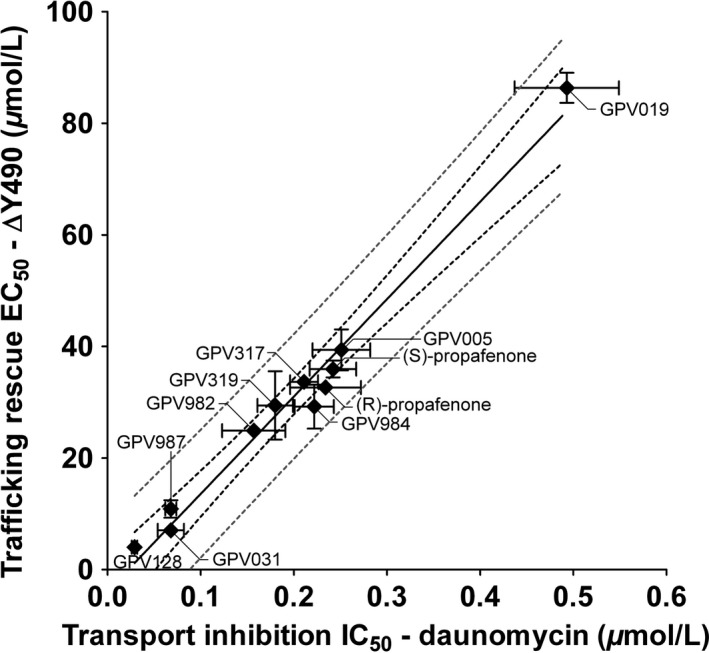
Correlation between EC_50_ values for trafficking rescue and IC_50_ values for daunomycin transport inhibition. The square of the correlation coefficient is 0.98 with a *P* < 0.001. A linear regression fit minimizing sum of squares, the 99% confidence interval (black dashed lines), and 99% prediction interval (grey dashed lines) are shown. Mean ± S.D. from at least two independent experiments.

As previously shown by photolabeling, propafenone‐binding sites are located at the TMD/TMD interface (Pleban et al. 2005) at a distance of more than 60 Å from the sites of mutation (Fig. S2). Propafenones are substrates of P‐gp and thus also act as competitive inhibitors of other substrates. Binding to active sites can thus be monitored by determining IC_50_ values. If binding to the active sites indeed mediated both the inhibitory and the pharmacochaperone effect of the compound class, a correlation between IC_50_ and EC_50_ values would be expected in a larger set of compounds. Therefore, 11 compounds, which were identified as folding correctors, were also evaluated for P‐gp inhibitory activity. Figure 6 shows the correlation for mutant ∆Y490. The *R*² value was found to be 0.98. Thus, it is safe to infer that the effect of propafenones to correct folding is mediated by their binding to active sites through a long‐range effect.

### Rescue of interface mutants with allosteric P‐gp modulator cis‐flupentixol

**Figure 7:**
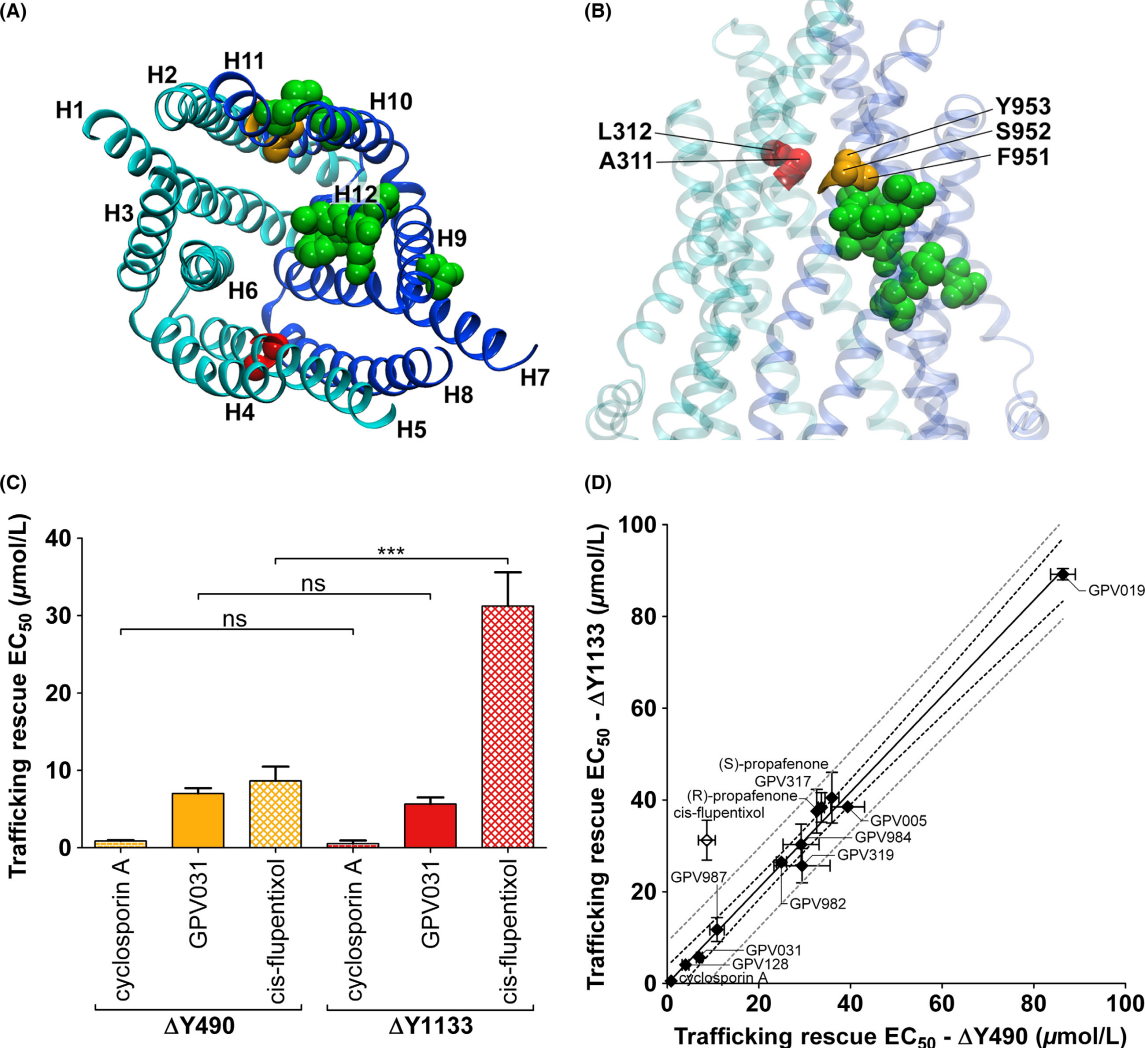
Homology model of human P‐gp (template M*. musculus* P‐gp (PDB ID: 4XWK)) indicating locations of residues involved in cis‐flupentixol binding (green) as well as *α*‐C‐atoms of residues at the TMD/TMD interface, which were previously found to be labeled by propafenones (red: A311, L312) (orange: F951, S952, Y953). The N‐terminal half of the protein is colored in cyan, the C‐terminal half is colored in dark blue. (A) Bottom view. (B) Side view. (C) Bar graph showing EC_50_ values for trafficking rescue of ∆Y490 (orange) and ∆Y1133 (red) with cyclosporin A, GPV031, and cis‐flupentixol. EC_50_ values were calculated by nonlinear regression analysis of concentration response curves. Means ± S.D. from at least three independent experiments were compared using one‐way ANOVA. Post hoc Tukey analyses were performed to find groups with significant different mean values. (D) Graph showing EC_50_ values for trafficking rescue of mutant ∆Y1133 in correlation with EC_50_ values for trafficking rescue of mutant ∆Y490. A linear regression fit minimizing sum of squares, the 99% confidence interval (black dashed lines), and 99% prediction interval (grey dashed lines) are shown. Mean ± S.D. from at least two independent experiments.

It has been demonstrated by several lines of evidence that substrates and several small molecule inhibitors bind to the TMD interface of P‐gp. The evidence is as follows: (1) refined structures of mouse P‐gp show the cyclic QZ peptide inhibitors in different positions along the interface. (2) Propafenones photolabel the TM helix 5/8 interface and in pseudosymmetric position helix 11, which contacts helix 2 of TMD1. (3) The majority of residues that have been identified in mutation analyses are predicted to be located at the interface in P‐gp homology models. Therefore, we tried to identify a compound, binding properties of which are restricted to one TMD only. The underlying hypothesis posited asymmetric rescue of such a compound. cis‐Flupentixol fulfills these criteria based on the amino acid residues in P‐gp, which are important for mediating the allosteric effects of cis‐flupentixol on substrate binding and ATPase activity (Mandal et al. 2012). A projection of these residues into a homology model of human P‐pg based on the mouse structure (Nicklisch et al. 2016) is shown in Figure 7A, B. These residues are exclusively located in TMD2. EC_50_ values for trafficking rescue were determined and found to be as follows: ∆Y490 8.7 ± 1.8 mol/L; mutant ∆Y1133: 31 ± 4.4 mol/L. Thus, the EC_50_ value is almost fourfold higher for the NBD2 than for the NBD1 mutant. This strongly contrasts with results obtained for propafenones and cyclosporin A (Fig. 7C, D).

## Discussion

Correct positioning of ICLs can be considered a prerequisite for engagement of the TMD coupling helices with membrane‐oriented NBD surfaces. This domain interaction leads to formation of the TMD/NBD transmission interfaces, which represent a critical element for the adoption of a fully folded conformation. Clustering of mutations at these interfaces is observed across different subfamilies and members of the human ABC protein family. The cystic fibrosis transmembrane conductance regulator CFTR is a chloride channel, deficiency of which gives rise to cystic fibrosis, the most frequently lethal inherited disease in humans. The paradigmatic ∆F508 mutation, which is observed in at least one allele in 90% of patients, conforms to mutant ∆Y490 in NBD1 of human P‐gp. Folding deficiency of this mutant has been identified as the root cause in this and a number of other mutants. The corresponding position of residue Y490 in NBD2 is Y1133 (Figs. 1, 2and S1A, B). Deletion of residue Y1133 compromises folding more severely than that of residue Y490 (Fig. 3C). Our finding is in accordance with earlier data by Loo and Clarke indicating that mutations in analogous positions of the two transmission interfaces affect P‐gp folding differentially and more severely, when mutations are located in NBD2 (Wang et al. 2007). In particular these authors demonstrated that coupling helices 2 and 3 and adjacent portions of ICLs 2 and 3 are more sensitive to point mutations than coupling helices 1 and 4 and adjacent portions of ICLs 1 and 4 (Loo et al. 2013; Loo and Clarke 2015).

Temperature shift experiments indicated that surface expression of both mutants was increased at lower temperature thus indicating the possibility for thermodynamic stabilization (Fig. 4). Several substrates/inhibitors of P‐gp can elicit a similar effect and therefore act as pharmacological chaperones. This has been shown previously for cyclosporin A, vinblastine, verapamil, and capsaicin (Loo and Clarke 1997, 1998, 2013, 2014; Loo et al. 2005). This evidence led us to explore the ability of the class of propafenone derivatives to act as pharmacological chaperones. Indeed, a number of compounds were able to increase surface expression in a concentration‐dependent manner with similar EC_50_ values for both mutants (Fig. 5). Our observations identified propafenone analogs as potent pharmacological chaperones with EC_50_ values of selected compounds in the low micromolar range.

The binding modes of propafenone analogs were previously characterized using photolabeling and mass spectrometry. These studies revealed two binding modes at the TMD interface, which are related to each other by pseudosymmetry (Pleban et al. 2005; Parveen et al. 2011). The two positively cooperative binding sites for rhodamine123 and Hoechst33342, named R‐ and H‐sites by Shapiro and Ling (Shapiro and Ling 1997) after their eponymous substrates, may relate to a preference for one of these two binding modes. The distance between the photolabeled active site residues in the apex of the central cavity and the positions of mutations at the transmission interface can be estimated in different crystal structures of ABC transporters and homology models of human P‐gp to be more than 60 Å (Fig. S2). We thus addressed the question, if a long‐range effect was responsible for folding correction. A correlation between IC_50_ values for transport inhibition and EC_50_ values was found for a larger set of propafenone analogs (Fig. 6). This correlation makes it likely that rescue is indeed brought about by a long‐range effect.

**Figure 8:**
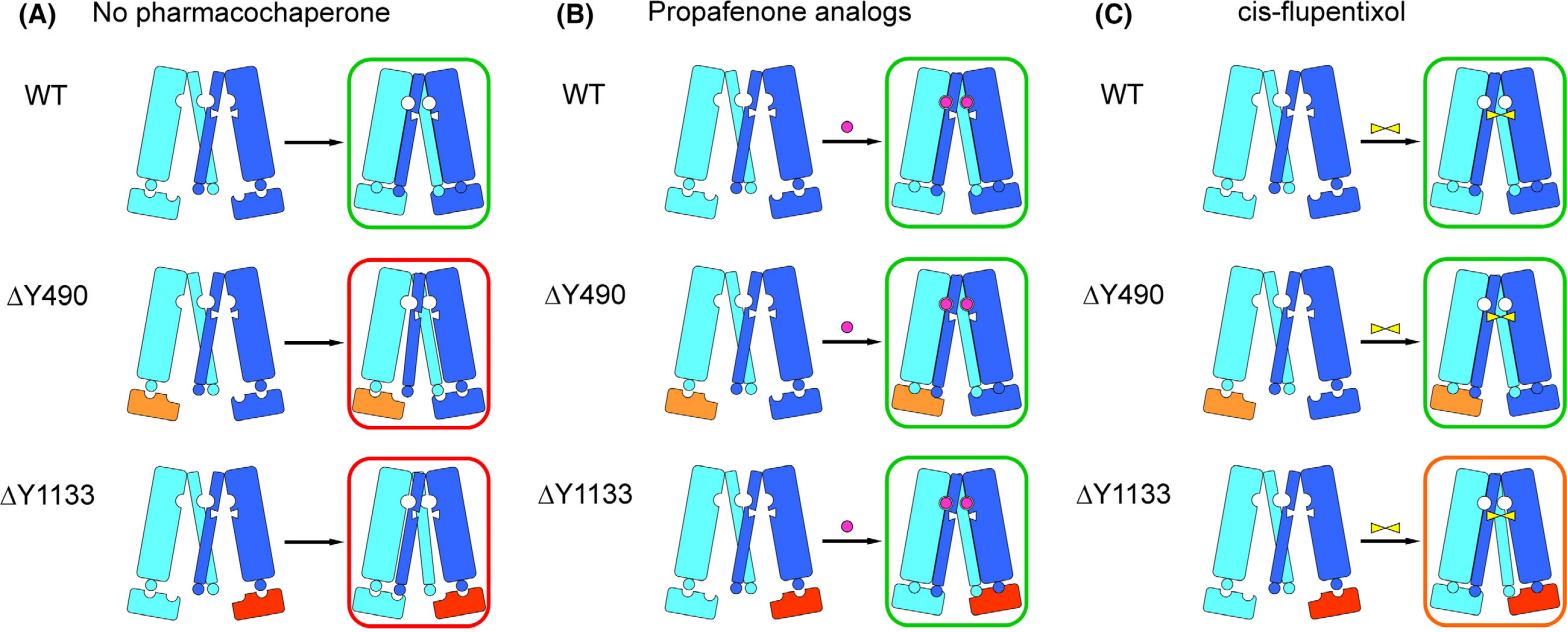
Cartoons displaying the posttranslational domain assembly of wild‐type P‐gp and transmission interface mutants ∆Y490 (NBD1: orange) and ∆Y1133 (NBD2: red). The N‐terminal half of P‐gp is colored in cyan, the C‐terminal half in dark blue. Starting points prior to domain assembly are depicted on the left and folding end points are shown on the right. (A) In the absence of pharmacochaperones only wild‐type P‐gp reaches a fully folded conformation (green box), whereas interface mutants remain outside the proteostasis boundary (red boxes). (B) Treatment with propafenone analogs (purple circles) results in a fully folded conformation in wild type and mutants (green boxes). (C) cis‐Flupentixol (yellow bow tie symbol) rescues folding of mutant ∆Y490 (green box), whereas mutant ∆Y1133 can only be rescued partially (orange box).

In order to achieve their native conformation, proteins have to undergo cotranslational and posttranslational folding. As shown for CFTR, proteins first attain a loosely folded conformation (Kleizen et al. 2005), which subsequently is compacted when interdomain interfaces are formed. This cooperative posttranslational domain assembly is required for stabilization of protein domains, absence of which represents a root cause for global misfolding and impaired trafficking ensuing from point mutations (Du and Lukacs 2009). Accordingly, deletion of residue F508 does not only affect the conformational stability of NBD1 but also disturbs the stability of TMD1, TMD2, and NBD2, as shown by limited proteolysis (Rabeh et al. 2012). Stabilization of TMDs through interaction with NBD1 is crucial for the rescue of CFTR mutant ∆F508 (Rabeh et al. 2012). Thus, cooperative domain folding is likely to also contribute to domain assembly of other ABC transporters (Du and Lukacs 2009). In light of this model we postulate the following mechanism for trafficking rescue of ABCB1 with active site compounds (Fig. 8): in the absence of pharmacological chaperones (Fig. 8A) only wild type attains its fully folded conformation (right, boxed in green), while the mutants reach an end point of folding, which lies outside the proteostasis boundary (boxed in red). In the presence of propafenones, folding of both mutants is corrected (Fig. 8B). cis‐Flupentixol rescues mutant ∆Y1133 to a lesser extent than mutant ∆Y490 as evidenced by an almost fourfold higher EC_50_ value (Fig. 7C, D). A partial rescue is accounted for by the cooperative nature of domain assembly (framed in orange) (Fig. 8C). Hence, mutation‐induced conformational changes in one domain compromise domain interplay on more general terms. Active site compounds contribute an energetic term by selecting conformations of an a priori flexible domain, which have an increased propensity to interact with their congeneric partner domain(s). In the case of ABC proteins and propafenones, this would result in an increased probability of one TMD to interact with the other. This model is consistent with the concept laid out in the conformational selection principle (Changeux et al. 1967; Burgen 1981). Mechanistically, this implies that small molecular weight ligands first interact with one TMD and this intermediate forms a ternary complex with the other TMD. The nascent conformation initially lacks cross‐domain interactions thus requiring much higher concentrations of compounds for pharmacochaperone activity than for transport inhibition. Indeed, the approximately 160‐fold higher concentration required for folding correction supports this notion (Fig. 6).

In contrast to competitive inhibitors, cis‐flupentixol has been reported to prevent translocation and subsequent dissociation of radiolabeled P‐gp substrate iodoarylazidoprazosin without competing for binding to the active sites (Maki et al. 2003; Maki and Dey 2006). Multiple sequence alignments of mammalian P‐gp homologues show conservation of about 60% of all TMD residues (Mandal et al. 2012). Dey and coworkers mutated nonconserved residues in the TMDs in order to identify residues involved in binding of cis‐flupentixol. These residues were found to be exclusively located in TMD2 (Fig. 7A, B) in contrast to those identified for a number of paradigmatic P‐gp substrates – including propafenones, verapamil, and vinblastine. We here report that the ∆Y490 mutant is rescued more efficiently than the ∆Y1133 mutant, which requires approximately fourfold higher pharmacochaperone concentration for rescue (Fig. 7C, D). cis‐Flupentixol interacts with residues located in TM helices 9, 10, 11, and 12. Formation of the binding site around the ligand stabilizes the positions of these helices, thereby defining the spatial orientation of ICL4. We posit that this stabilization of the helical bundle of TMD2 promotes interaction with NBD1. Folding correction of the NBD2 mutant occurs at much higher compound concentrations and is likely due to a global stabilization effect.

In conclusion, the difference in the rescue of P‐gp interface mutants with cis‐flupentixol reflects the asymmetric binding properties of this P‐gp modulator. In contrast, several different active site compounds aid in correct positioning of both intracellular loops. Thereby, they promote the engagement of coupling helices by a groove, which is present on the surface of either of the two NBDs and which is oriented toward the TMD. Correct folding of the NBDs is required for proper positioning of ICLs and, therefore, the NBDs act as proteinaceous chaperones in the folding of the wild‐type protein. Conversely, the TMDs also stabilize the NBD dimer. In the case of NBD misfolding the likelihood of ICLs and TMDs to adopt their fully folded conformation is decreased due to the loss of NBD‐assisted positioning. In the latter case, active site compounds aid in correct positioning of intracellular loops, thereby enabling the protein to reach a conformation which is required to pass folding and trafficking checkpoints.

## Author Contributions

Participated in research design: Spork, Chiba. Conducted experiments: Spork, Sohail, Stockner. Contributed new reagents or analytic tools: Schmid, Ecker. Performed data analysis: Spork, Chiba, Stockner. Wrote or contributed to writing of the manuscript: Spork, Freissmuth, Chiba.

## Disclosure

None declared.

## Supporting information


**Figure S1.** The positions of tyrosine 490 in NBD1 (orange), tyrosine 1133 in NBD2 (red), and coupling helices (CH) of ICLs2 and 4 (CH2, CH4) are depicted in a homology model of human P‐pg (template *M. musculus* P‐gp (PDB ID: 4XWK)).

**Figure S2.** Positions of residues Y490 (orange) and Y1133 (red) as well as residues, which were previously found to be photolabeled by intrinsically photoactive propafenone analogs are indicated in a homology model of human P‐pg (template *M. musculus* P‐gp (PDB ID: 4XWK)).

**Table S1**. SNPs in ICLs and NBDs of human ABC proteins.
