# Supplementary figures and images for: Folding correction of ABC‐transporter ABCB1 by pharmacological chaperones: a mechanistic concept
Source: Pharmacol Res Perspect. 2017 May 26;5(3):e00325. doi: 10.1002/prp2.325 (PMC5464349; doi:10.1002/prp2.325)

**Figure S1A**

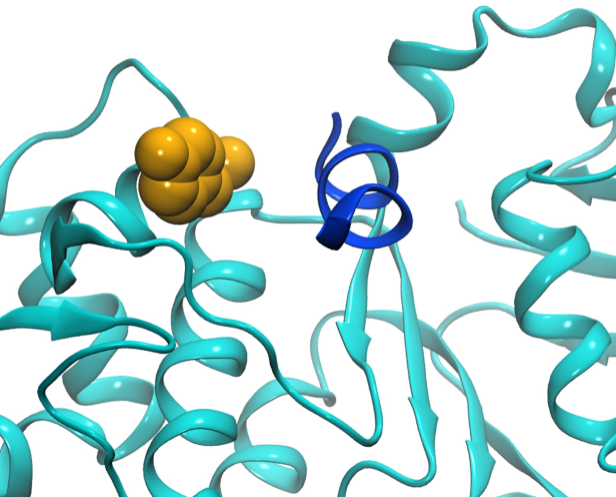

Supplement: Supplementary file 1 — Figure S1. The positions of tyrosine 490 in NBD1 (orange), tyrosine 1133 in NBD2 (red), and coupling helices (CH) of ICLs2 and 4 (CH2, CH4) are depicted in a homology model of human P‐pg (template M. musculus P‐gp (PDB ID: 4XWK)). [file PRP2-5-e00325-s001.pdf]

**Figure S1B**

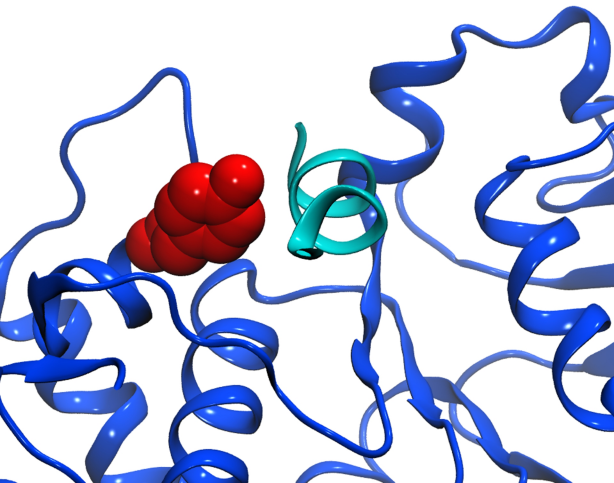

Supplement: Supplementary file 2 [file PRP2-5-e00325-s002.pdf]

**Figure S2**

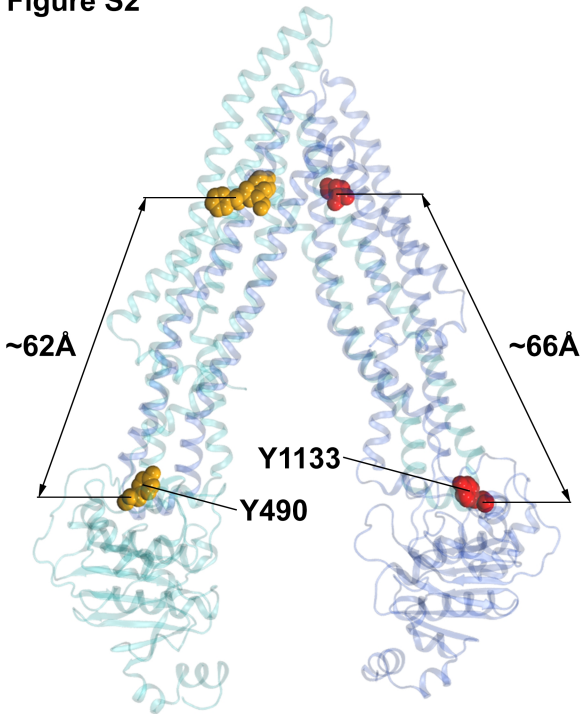

Supplement: Supplementary file 3 — Figure S2. Positions of residues Y490 (orange) and Y1133 (red) as well as residues, which were previously found to be photolabeled by intrinsically photoactive propafenone analogs are indicated in a homology model of human P‐pg (template M. musculus P‐gp (PDB ID: 4XWK)). [file PRP2-5-e00325-s003.pdf]
