# Supplementary material for: Folding correction of ABC‐transporter ABCB1 by pharmacological chaperones: a mechanistic concept
Source: Pharmacol Res Perspect. 2017 May 26;5(3):e00325. doi: 10.1002/prp2.325 (PMC5464349; doi:10.1002/prp2.325)
Supplement: Supplementary file 4 — Table S1. SNPs in ICLs and NBDs of human ABC proteins. [file PRP2-5-e00325-s004.docx]

**Supplemental Data**

**Folding correction of ABC-transporter ABCB1 by pharmacological chaperones: a mechanistic concept**

Matthias Spork^1^, Muhammad Imran Sohail^1,5^, Diethart Schmid^3^, Gerhard F. Ecker^4^, Michael Freissmuth^2^, Peter Chiba^1*^, Thomas Stockner^2^

**
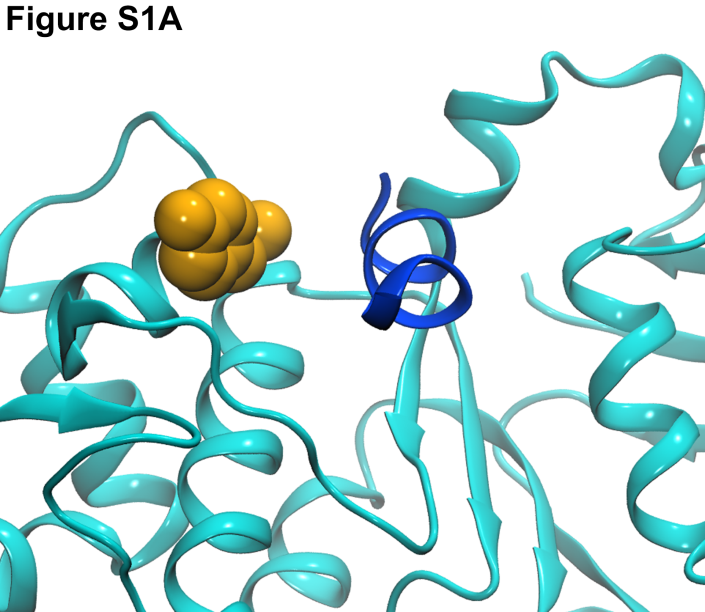
**

**
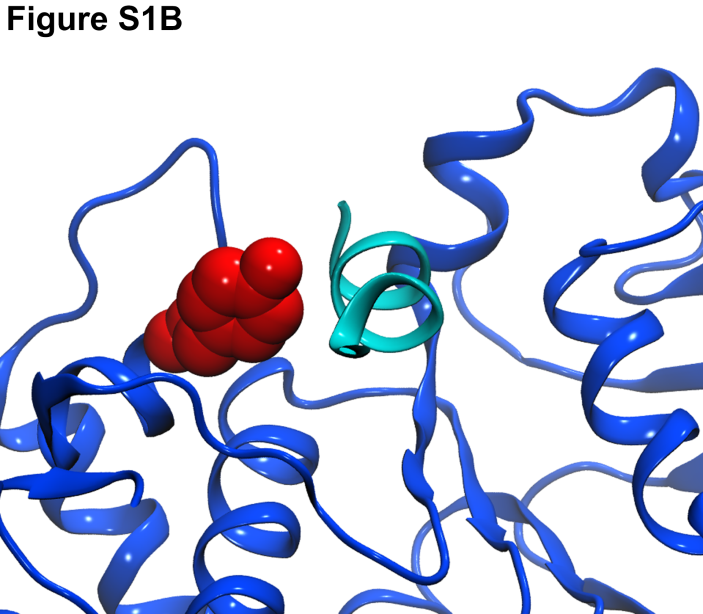
**

**Figure S1:** The positions of tyrosine 490 in NBD1 (orange), tyrosine 1133 in NBD2 (red) and coupling helices (CH) of ICLs2 and 4 (CH2, CH4) are depicted in a homology model of human P-pg (template *M. musculus* P-gp (PDB ID: 4XWK)). The N-terminal half of the protein is colored in cyan, the C-terminal half is depicted in dark blue. **A:** Side view (NBD1, CH4). **B:** Side view (NBD2, CH2).

**
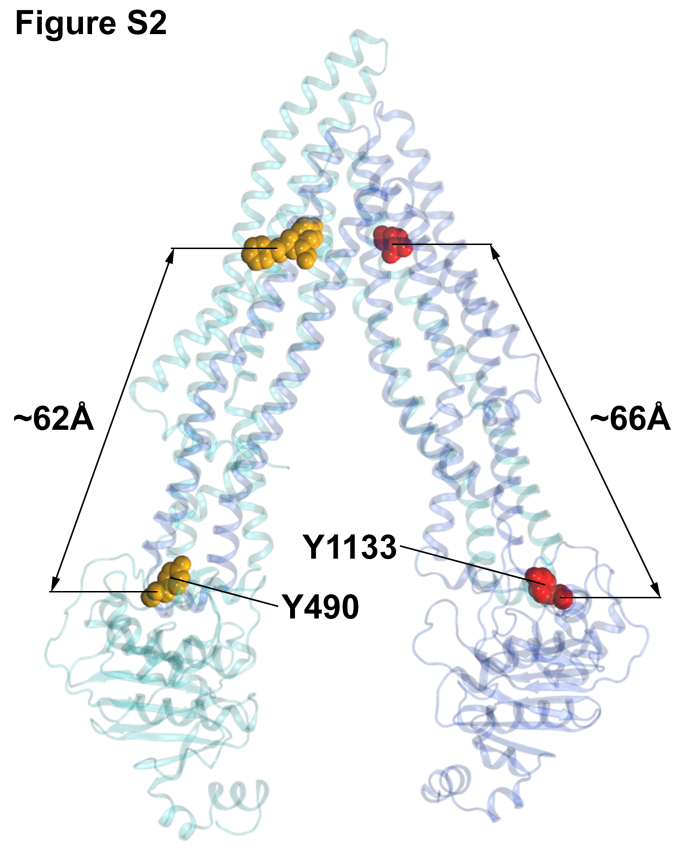
**

**Figure S2:** Positions of residues Y490 (orange) and Y1133 (red) as well as residues, which were previously found to be photolabeled by intrinsically photoactive propafenone analogs are indicated in a homology model of human P-pg (template *M. musculus* P-gp (PDB ID: 4XWK)). The latter are located at the TMD domain interface (red: A311, L312) (orange: F951, S952, Y953). The N-terminal half of the protein is colored in cyan, the C-terminal half is depicted in dark blue.

**Supplementary Table 1: SNPs in ICLs and NBDs of human ABC proteins**

References for listed SNPs can be found in the ABC mutation database (<http://abcmutations.hegelab.org>)

| ABC protein | SNPs | analogous residue in ABCB1 | motif |
| --- | --- | --- | --- |
| ABCA1 | N935S | S429 | NBD1 - Walker A |
| ABCA1 | A1046D | A544 | NBD1 - signature motif |
| ABCA1 | R1068H | E566 | NBD1 |
| ABCA1 | R1925Q | D1049 | NBD2 |
| ABCA1 | R2004K | A1128 | NBD2 - 1^st^ alpha helix |
| ABCA1 | A2028V | F1157 | NBD2 |
| ABCA1 | R2081W | K1212 | NBD2 |
| ABCA3 | N568D | S429 | NBD1 - Walker A |
| ABCA3 | L1553P | V1214 | NBD2 |
| ABCA3 | Q1591P | R1250 | NBD2 |
| ABCB1 | G185V | G185 | ICL1 |
| ABCB1 | S893A | S893 | ICL4 |
| ABCB1 | I1196S | I1196 | NBD2 - Walker B |
| ABCB4 | I541F | I539 | NBD1 - signature motif |
| ABCB11 | E186G | E159 | ICL1 |
| ABCB11 | L198P | L171 | ICL1 |
| ABCB11 | E297G | Q270 | ICL2 |
| ABCB11 | L413W | P385 | NBD1 |
| ABCB11 | R432T | R404 | NBD1 - A-loop |
| ABCB11 | K461E | K433 | NBD1 - Walker A |
| ABCB11 | D482G | D454 | NBD1 |
| ABCB11 | N490D | N462 | NBD1 |
| ABCB11 | A570T | A542 | NBD1 - signature motif |
| ABCB11 | T655I | T627 | NBD1 |
| ABCB11 | R1128H | R1085 | NBD2 |
| ABCB11 | R1153C | R1110 | NBD2 |
| ABCB11 | R1268Q | R1225 | NBD2 |
| ABCC2 | W709R | V478 | NBD1 |
| ABCC2 | R768W | R538 | NBD1 - signature motif |
| ABCC2 | S789F | S559 | NBD1 - D-loop |
| ABCC2 | I1173F | V907 | ICL4 - IH4 |
| ABCC2 | R1174H | V908 | ICL4 - IH4 |
| ABCC2 | R1181L | K915 | ICL4 |
| ABCC2 | ∆R1392 | A1128 | NBD2 - 1^st^ alpha helix |
| ABCC2 | ∆M1393 | E1129 | NBD2 - 1^st^ alpha helix |
| ABCC2 | A1450T | A1189 | NBD2 - signature motif |
| ABCC6 | R1138Q | V908 | ICL4 - IH4 |
| ABCC6 | R1314W | R1085 | NBD2 |
| ABCC6 | G1321S | G1092 | NBD2 |
| ABCC6 | R1339C | R1110 | NBD2 |
| ABCC7 | G480C | G449 | NBD1 |
| ABCC7 | ∆I507 | R489 | NBD1 - 1^st^ alpha helix |
| ABCC7 | ∆F508 | Y490 | NBD1 - 1^st^ alpha helix |
| ABCC7 | S549R(N) | S532 | NBD1 - signature motif |
| ABCC7 | A559T | A542 | NBD1 - signature motif |
| ABCC7 | S945L | T785 | ICL3 |
| ABCC7 | D979A | D821 | ICL3 |
| ABCC7 | H1054D | I896 | ICL4 |
| ABCC7 | G1061R | N903 | ICL4 |
| ABCC7 | L1065P | V907 | ICL4 - IH4 |
| ABCC7 | R1066C | V908 | ICL4 - IH4 |
| ABCC7 | L1077P | M919 | ICL4 |
| ABCC7 | H1085R | P927 | ICL4 |
| ABCC7 | N1303K | N1130 | NBD2 - 1^st^ alpha helix |
| ABCC8 | R495Q | K249 | ICL2 |
| ABCC8 | E501K | E255 | ICL2 |
| ABCC8 | L503P | V257 | ICL2 |
| ABCC8 | F686S | F399 | NBD1 |
| ABCC8 | G716V | G430 | NBD1 - Walker A |
| ABCC8 | G1342E | G1033 | NBD2 |
| ABCC8 | L1349Q | V1040 | NBD2 |
| ABCC8 | ∆F1387 | V1079 | NBD2 |
| ABCC8 | M1394R | F1086 | NBD2 |
| ABCC8 | R1418H | R1110 | NBD2 |
| ABCC8 | D1471H | S1166 | NBD2 |
| ABCC8 | R1493W | R1188 | NBD2 - signature motif |
| ABCC8 | L1543P | Q1238 | NBD2 |
| ABCG2 | K86M | K433 - K1076 | NBD - Walker A |
| ABCG2 | Q141K | R489 - A1132 | NBD - 1^st^ alpha helix |
| ABCG2 | T153M | E501 - V1146 | NBD |
| ABCG2 | I206L | I551 - I1196 | NBD - Walker B |
| ABCG2 | F208S | L553 - L1198 | NBD - Walker B |
